# Medico-Legal Trial: Disputed Will—Plea of Mental Incapacity Skipper and Skipper *v*. Bodkin and Others

**Published:** 1860-01-01

**Authors:** 


### MEDICO-LEGAL TRIAL.—DISPUTED WILL.—PLEA OF MENTAL INCAPACITY.

### COURT OF PROBATE, December 8.

Before Sir C. Cresswell and a Special Jury.

SKIPPER AND SKIPPER *v.* BODKIN AND OTHERS.

Mr. Sergeant Pigott, Dr. Tristram, and Mr. Couch were counsel for the plaintiffs; Mr. Edwin James, Q.C., Dr. Phillimore, Q.C., and Mr. Coleridge for the defendants.

The plaintiffs in this case, John Skipper and William Skipper, are the executors of the will of the Rev. William John Smyth, of Cringleford, near Norwich, who died on the 23rd of April last. The defendants are John James Bodkin and William Thomas Bodkin, the nephews, and Mrs. Macdermott, the niece of the deceased; and they opposed probate of the will propounded by the plaintiffs on the grounds that at the time of its execution the deceased was not of testamentary capacity, and that he was induced to execute it through the undue influence of the plaintiffs and of other persons.

The deceased was the son of Colonel Smyth, and the last representative of an old Gloucestershire family. He had one sister, who many years ago married Captain, afterwards Major, Bodkin, and upon his death married a surgeon, also named Bodkin. Mr. Smyth was educated at Emmanuel College, was ordained, and held a curacy until 1812. In 1808 his father, Colonel Smyth, died, and he came into possession of landed property of the value of from 1500*l.* to 2000*l.* a-year. He lived from 1812 until his death at Cringleford, in Norfolk, seeing little or no society. Mrs. Smyth, his mother, lived with him, but she died in 1834, and his property was then increased to the extent of about 7000*l.* In 1832 he made a will, by which he devised the family property to his sister, Mrs. Bodkin, and the property which had been purchased to a Mr. H. Gilbert, a medical man at Norwich, with whom he was on terms of intimacy. A few years afterwards he gave Gilbert an annuity of 100*l.*, and in 1836 executed a deed of gift to him of landed property worth about 3000*l.* Gilbert about that time married, gave up his intimacy with Mr. Smyth, and resumed the practice of his profession. Mr. Smyth then became intimate with Mr. Alfred Massey, the son of a brewer at Norwich, paid the expense of sending him to college, and announced his intention of making him his heir. In 1838 Mrs. Bodkin died, and her death was announced to Mr. Smyth by her son. Mr. Smyth continued his friendship for Massey until 1848, and made various wills in which he was named residuary legatee of the real estate. About 1848 Massey went to live at some distance from Cringleford. Mr. Smyth then became intimate with the family of the Delfs, which had occupied farms on the estate for a great number of years. He became very friendly with Samuel Delf, and continued that friendship up to the time of his death. In 1854 and 1855 he was also on intimate terms with a Mr. Newton, and executed deeds of gift and wills in his favour. On the 16th July, 1857, he went to the office of his attorneys in Norwich, Messrs. Skipper and Sons, and gave instructions for a fresh will. A will was accordingly drawn up in pursuance of those instructions, and was executed by Mr. Smyth on the 31st of the same month. Its effect was to appoint Massey residuary legatee of his real estate, charging it with an annuity of 50*l.* to Mr. and Mrs. Edwards, his servants, for their joint lives and for the life of the survivor, to confirm and ratify the various deeds of gift that he had previously executed, and to appoint John Skipper and William Skipper his executors, giving them legacies of 50*l.* each. This was the will now propounded. The attesting witnesses were Dr. Hutchison, his medical attendant, and a Mr. Dunnan. It is necessary to mention that in all the various deeds of gift, except that to Gilbert, a condition was inserted purporting to reserve the rents to the donor for his life, and as the donees did not enter into possession, he continued to receive the rents, amounting to about 1800*l.* a year, until his death, although he had by that time conveyed away the whole of his property. He died on the 23rd of April, 1859, aged eighty-one.

Mr. John Skipper, an attorney who had practised at Norwich for the last 47 years, said he had been acquainted with Mr. Smyth for 40 years, and had acted as receiver to his estates from 1829 until his death. The amount of the rents and the investments on mortgage varied from 1700*l.* to 2000*l.* a-year. On the death of his mother, Mrs. Smyth, in 1834, an annuity of 300*l.* a-year fell in. Mrs. Smyth left a legacy to Mrs. Bodkin, and made Mr. Smyth her residuary legatee. Mr. Smyth was a great will maker, and he had prepared wills for him in 1832, 1834, and 1835. Major Bodkin and his wife stayed at Cringleford for a few days in 1834 to settle the affairs relating to Mrs. Smyth's will. Mr. Smyth always manifested a great dislike to the Bodkins, and in 1842 he gave instructions for a will by which they should be expressly excluded. He executed a will in that year by which he left the bulk of his real property to Mr. Alfred Massey, gave 3000*l.* to witness and his sons, and legacies to various other persons. In 1844 he executed another will, by which he gave the real estate to Alfred Massey in tail, with remainder, in default of issue, to witness, and confirmed the bequest of 3000*l.* to him and his sons. By a will of 1846 he left the property to witness and his son in trust for the use of Massey, alleging as a reason that Massey was rather given to gambling, and was not competent to manage it. Upon Massey's marriage, in 1846, he executed a deed of gift of a farm to him, and in August of that year, by codicil, substituted a legacy of 1000*l.* for the 3000*l.* previously given to witness and his sons. Of this alteration Mr. Skipper gave the following explanation:—He had 2000*l.* in his hands belonging to Mr. Smyth, and he proposed that he should take the money then, instead of waiting until Mr. Smyth's death, and give Mr. Smyth an agreement to pay him interest upon it during his life. Mr. Smyth concurred in this arrangement, and the agreement was given. Mr. Smyth had lent 500*l.* each to two of his sons, and subsequently he released them from this debt, but revoked the bequest of the remaining 1000*l.* Thus the entire legacy was revoked, and he received the interest on the 3000*l.* until he died. In 1847 he made another will, giving a farm to Samuel Newton Delf. In September, 1848, there was a codicil executed, devising to the same person a further portion of the estate. By a codicil of December, 1849, a farm was given to Dr. Hutchison; by one of July, 1850, another farm was given to William Skipper, in lieu of one given by a previous will, and then transferred to Delf; in December, 1850, an annuity of 100*l.* was given to Dr. Hutchison, but was afterwards revoked. Massey still continued a residuary legatee. In 1841 Mr. John James Bodkin, the son of Major Bodkin, went to see his uncle. He had written to announce his visit, and Mr. Smyth replied that he lived a very retired life, but Mr. Bodkin might come for three days. He accordingly stayed at Cringleford for that time. In 1855 Mr. Bodkin came to Mr. Skipper's office, and said he wished to learn the particulars of some difficulties in which his uncle had become involved. Mr. Skipper told him about the different wills and conveyances he had executed, and that nothing had been left to the Bodkin family. Mr. Bodkin said he never expected anything, and that he had conversed with his uncle for two hours, and he was quite competent to take care of himself, In 1854 Mr. Smyth gave Mr. Skipper instructions to convey another farm to Delf. Mr. Skipper advised him not to execute any more conveyances, and refused to act upon his instructions, whereupon Mr. Smyth took offence, and although Mr. Skipper continued to receive his rents, he went for about 18 months to another attorney. Mr. Skipper considered him perfectly competent to make a will in July, 1857. He was never paid any commission for receiving the rents, but he had the usual fees as steward of the manor, and charged for his professional business.

Cross-examined by Mr. E. James.—The round value of the estate given to my son was 3000*l.* I prepared the conveyance. My son mortgaged it for 1900*l.* It was given by Mr. Smyth out of friendly feeling. Delf was 21 or 22 years of age when he first made acquaintance with Mr. Smyth. Massey was about the same age. Newton was between 20 and 30, and was the son of a well-known land surveyor at Norwich. I did not visit Mr. Smyth, because I could not descend to the level of his character and habits. I heard that he was fond of licentious and lascivious conversation, but he never said anything improper in my presence. The value of the estate was about 60,000*l*, and he had disposed of all of it by the time of his death, but he continued to receive the rents. Gilbert was a surgeon, the son of a magistrate for the county. He sold the estate that Mr. Smyth gave him about three years after it came into his possession for 3300*l.* Gilbert had lived at his house for two or three years. Mr. Smyth was a man of very eccentric habits. His eccentricity consisted in withdrawing himself from the society of persons of his own station and associating with persons of inferior education. He never complained to me of bills of exchange having been obtained from him. He told me that he had borrowed 8000*l.* on mortgage, for the purpose of purchasing a farm which Delf had occupied, and that Delf had obtained possession of it. He said Delf had promised to secure 200*l.* a-year to him, but had not done so, and by his instructions I drew up a deed for the purpose of securing it, but I could not get Delf to sign it. Delf paid the interest on the mortgage. Mr. Smyth also told me on one occasion that Delf and Newton had got possession of the family plate, and I applied for it, but Delf produced a memorandum signed by Mr. Smyth, and I was not able to recover it. That was towards the end of 1856. Mr. Smyth also complained that Massey had taken away the title-deeds of Cringleford without his consent. I wrote to Massey for them, and he replied that they were not in his possession, but in the hands of a mortgagee. In 1857, Massey brought an action against him on a bill of exchange. I pleaded to the action, and in the affidavit which it was necessary to make in order to obtain leave to plead, I said that Mr. Smyth had been subject to long attacks of debilitating illness, and his memory was so impaired that he could not give information as to facts which had long ago happened. Mr. Smyth was liable to be imposed on and led away by importunities. The reason he gave for the voluntary conveyances that he executed was that the persons he wished to benefit had contributed to his comfort. I frequently asked him about the conveyances, because I thought he might be acting under threats of some kind, but he always denied it.

Mr. William Skipper, the son of the last witness, was examined and cross-examined at considerable length with regard to the various transactions of which his father had spoken. He stated that on the 16th of July, 1857, Mr. Smyth had come to his office and given instructions for another will. The will was prepared, and he called again on the 23rd, but when it was read over he suggested an alteration as to the manner in which the legacies to the Edwardses were to be paid. It was therefore necessary to re-copy the will, and he made an appointment for the 31st to execute it. The witness was not present at the execution, but said that Mr. Smyth was perfectly competent to understand the effect of what he was doing. He further said that in 1852 Mr. Smyth conveyed an estate to him which he mortgaged in the following year for 1900*l*. In 1855 Mr. Smyth wished to raise some money, and the estate was sold, the proceeds, after payment of the mortgage, going to Mr. Smyth. By a codicil of January, 1857, to the will of 1854, Mr. Smyth revoked a will he had made between 1854 and 1857 in favour of Newton, revoked the residuary bequest in the will of 1854, and confirmed all conveyances previous to that date.

Mr. James Stark Skipper, a brother of the last witness, was present at the signature of the will, and proved its due execution. He also stated that early in 1856 it was discovered that Mr. Smyth had drawn out the whole amount of the rents deposited at his banker's a few days after the audit, and suspecting that he had been induced to sign blank checks, Messrs. Skipper proposed that all the payments and receipts should pass through their hands, and that he should receive 20*l*. a month for small expenses. That arrangement was carried out and continued up to his death. His explanation of the matter was that his name had been forged, but it was thought more probable that he had signed checks and forgotten it.

Dr. Hutchison said that he had attended Mr. Smyth since 1836, and was one of the attesting witnesses to the will. He was of perfectly competent understanding, and he said the will had been read over to him and it expressed his wishes. He had for many years suffered from hernia. He was of nervous temperament and anxious about his health. In 1853 he conveyed a farm of 36 acres to Mrs. Hutchison, but he received the rents during his life. When he spoke of the Bodkins he said they should have none of his property, because Major Bodkin had insulted him.

Cross-examined.—Mr. Smyth was a quiet, retired man, but he was fond of loose conversation. Mrs. Hutchison did not visit him, because there were rumours about his habits which made it improper for ladies to go to his house. For some time he paid a regular sum, 5*l*. a-quarter, instead of fees, for medical attendance, but at the beginning of 1857 he said he could not afford that sum, and proposed to pay in future by fees. For the next six months the bill for medical attendance amounted to 64*l*., as they were almost daily visits. Mr. Smyth objected to the charge, and in August, 1857, he ceased to attend him. Mr. Smyth had in previous years given him various sums of money, which he looked upon as presents, but which were due for medical attendance, but never more than 100*l*. at a time. In November, 1856, he attested the execution of a will at Massey's house. (By this will the bulk of the property was left to Massey.) He also attested the codicil of January, 1857, executed at his own house, and prepared by Mr. Skipper. Mr. Smyth was a man of eccentric habits, fond of playing on the piano, and he could run over the keys in a masterly manner, but could only play one tune—"God save the Queen." He would sometimes show off his agility by dancing and running. He had heard from Mr. Smyth of a chargeof indecent assault that was made against him several years ago. He never knew until after Mr. Smyth's death that a farm had been left him by one of the wills.

Robert Dunnan, accountant to the Norwich Equitable Fire Office, and the other attesting witness, also proved the execution of the will. Up to 1853 he had kept Mr. Smyth's accounts, as he was unable to add up three figures. He could not understand the effect of wills and conveyances unless they were explained to him. Witness had often cautioned him against Newton and the Delfs, and said they would reduce him to a wheelbarrow. No person of any respectability associated with him. He had told witness that he intended to leave him 200*l.*, but he had always refused to accept it. His reason was that he did not wish to be classed with the young men with whom Mr. Smyth associated.

Mr. H. B. Miller, an attorney, gave an account of the mortgage transactions in which Delf and Mr. Smyth had been engaged, and said that Mr. Smyth was quite competent to understand them.

Cross-examined.—In 1855 or 1856 he gave the Rev. H. Delfosse, Dissenting minister, a small farm of about 1000*l.* value, upon which witness in the follow-year advanced 600*l.*

The Court adjourned at the conclusion of this witness's examination.

*December 9th.*

At the sitting of the Court the examination of witnesses in support of the will was resumed.

Mr. Hardy, a grocer of Norwich, said the testator had dealt with him for many years, and used to drive or walk to the shop, give his orders, and pay his bills. There did not appear to be any defect in his understanding. He did not pay his own bills after 1856.

Mr. Cannell, an overseer of Cringleford and collector of the poor-rate, proved that he was generally paid by Mr. Smyth. Five or six years ago he wrote a check for 90*l.* instead of 19*l.*, but discovered his mistake and wrote another. In the quarter before he died he complained that the rate was rather high, and said, "D— the poor, if I had my own mind, they should be sent to their own parishes." He added that he would rather give his money to them than to his relatives. On a previous occasion he said he would rather burn his money than let his relatives have it.

Cross-examined.—Alfred Massey is now living at Cringleford. He and I have been out shooting together once, but we are not on intimate terms. Sometimes Mr. Smyth did not know me when I first went in. Either Edwards or Mrs. Edwards was with him when he paid the rates during the last three or four years of his life.

Mr. Steward, a solicitor, who had advanced 8000*l.* on an estate called Topcroft, in 1854, proved that Mr. Smyth had executed the mortgage and given authority to Delf to receive the money.

Mr. Swatman, a solicitor at Norwich, who had been engaged in the same transaction on the part of Mr. Smyth and Mr. Delf, said that Mr. Smyth told him the money was to be given to Delf, and Delf was to pay the interest. He had not the slightest doubt of Mr. Smyth's competency.

Mr. White, a London attorney, who had prepared and attested the will of 1856 in favour of Massey, stated that he had received his instructions from Mr. Smyth, who expressed his confidence in Mr. Massey, and seemed perfectly to comprehend the business.

The attesting witnesses to a number of the various wills and codicils were also called, and gave their opinions that Mr. Smyth was of testamentary capacity.

Several letters written by Mr. Smyth to Mr. Massey and to Mr. Skipper were read, extending from 1849 to 1855, containing nothing at all remarkable either in matter or in manner. Two receipts, with his signature attached, dated the 22nd of February, 1859, and 24th March, 1859, for the monthly 20*l.* paid him by Messrs. Skipper, were also put in evidence.

Mr. Edwin James said that no witnesses would be called for the defendants.

Mr. Serjeant Pigott therefore summed up the plaintiff's evidence. He submitted that as there was no evidence at all of undue influence, the only question was that of incompetency. Even if the jury thought that the testator was a man of immoral habits and liable to let his associates take advantage of him, that was no reason for invalidating his will. His disposition of his property might appear whimsical, but he had no relatives about him. He was alone in the world. He assigned reasons more or less valid for the benefits that he conferred on different persons, and it clearly appeared to be his intention that none of the Bodkins should have his property if he could help it. Various calumnies as to his mode of life had been insinuated in the course of the case, but they were not consistent with his letters and his acts, and no evidence had been given to show what foundation for them, if any, existed. There was nothing unusual or improper in the manner in which the instructions for the final will were given, or in which the will itself was executed, and both at the time of the execution and subsequently he gave indications of perfect testamentary capacity. It was several months afterwards that he objected to the charge of Dr. Hutchison and employed another medical man. The learned serjeant concluded by calling upon the jury not lightly to set aside a will, as they were all interested in upholding wills unless it could be clearly proved that they did not carry out the intentions of the testators.

Mr. Edwin James, in his address to the jury for the defendants, said that the ground upon which the will was opposed was, that this weak-minded and vacillating old man, not being of mental capacity to resist the dark and mysterious influence of his associates, Massey, Newton, and Delf, made various conveyances to them at different times, and confirmed all these conveyances by the will, not knowing that it would have that effect. The object of Messrs. Skipper in inducing him to execute it was to obtain the entire control, as executors, of the whole property, thinking there would be no one to call them to account. Mr. Bodkin, who was a gentleman of landed property in Ireland, and had represented Galway in Parliament, learnt in 1855 that his uncle was perfectly incompetent, and had got rid of all his property; but, instead of annoying the old gentleman by issuing a commission in lunacy, he took the proper course of waiting until he died, to see whether Messrs. Skipper would dare to set up such a will as the present. The learned counsel then went through the evidence, arguing that it proved that the old man was a mere automaton in the hands of Messrs. Skipper, or of any one who could get hold of him. He signed whatever was given to him, and became at last so utterly incapable of managing his own affairs, that he was only allowed a miserable 240*l.* a-year out of his 1800*l.*, and all his concerns, even the most trivial, were managed by Messrs. Skipper. He had accused Massey and Delf, the one of stealing his plate and the other of stealing his deeds; he had called Newton a rascal, and yet all his conveyances were confirmed by a compendious line in his final will. His conveyances to various people—Alfred Massey, William Massey, a Mr. Tyler and his wife, Hutchison's wife, Delf, and his relative the Rev. H. Delf, and others—comprised property to the value of no less than 60,000*l.*, and the will confirmed them all. The learned counsel commented in severe terms upon the conduct of Messrs. Skipper in their dealings with Mr. Smyth. They had, he said, the greatest interest in the result of this case, and it was their evidence alone which was relied on to uphold the will, because they had neither dared to call the Edwardses, who knew more about the deceased than anybody else, nor Delf, nor Massey, nor any of his intimate associates. In conclusion he expressed a hope that the jury would defeat the attempt now made to establish the validity of these suspicious transactions.

The learned Judge then summed up. With regard to the first issue he directed the jury that the deceased would not be incapable of making a will if he was able to understand the nature of the property he was disposing of, to bear in mind his relatives, and the persons connected with him, and to make an election as to the parties he wished to benefit. It was not enough, on the one hand, that he should be able to say "yes" or "no" to a simple question; nor, on the other hand, was it necessary that he should be a well-informed man or a scholar. He might be stupid, dull, or ignorant; but if he understood the nature of his property, and could select the objects of his bounty, that would be sufficient. With respect to the second question, in order to establish undue influence it was not enough to show that a man had been persuaded or cajoled by pretended friendship, nor that he had been induced by importunities or requests made from time to time. If that were sufficient, in how many families would wills be set aside! The influence of attachment, argument or importunity, would not suffice, unless the importunity was carried to such an extent that it amounted to depriving the testator of his free judgment, and of the exercise of his free will. If the will was the will he wished to make, by whatever means he might have been induced to make it, or by whatever persuasion, barring fraud, it would be a good will; but, if he made it in consequence of pressure, it was no longer his will, and must be set aside. With regard to Mr. Skipper, his Lordship observed that he would unquestionably have stood better before the public if he had not been so largely benefited by this unfortunate old man during his life. No doubt Mr. Smyth, early in his career, had been driven from society by some grievous imputation; and Mr. Skipper said that, in consequence of that stigma on his character, he did not visit him. Mr. Skipper would have done well to follow the example of Mr. Dunnan and refuse to take any benefit from him. His Lordship then read over the material parts of the evidence.

The jury retired at half-past five o'clock, and returned at the end of an hour and a half with a verdict for the plaintiffs upon the issue of capacity, and for the defendants upon the issue of undue influence.

